# Role of Functional Imaging Techniques to Assess Motor and Language Cortical Plasticity in Glioma Patients: A Systematic Review

**DOI:** 10.1155/2019/4056436

**Published:** 2019-11-11

**Authors:** S. Cirillo, M. Caulo, V. Pieri, A. Falini, A. Castellano

**Affiliations:** ^1^Neuroradiology Unit and CERMAC, Vita-Salute San Raffaele University and IRCCS San Raffaele Scientific Institute, Milan 20132, Italy; ^2^Department of Neuroscience, Imaging and Clinical Sciences and ITAB-Institute of Advanced Biomedical Technologies, University “G. d'Annunzio”, Chieti 66100, Italy

## Abstract

Cerebral plasticity is the ability of the central nervous system to reorganize itself in response to different injuries. The reshaping of functional areas is a crucial mechanism to compensate for damaged function. It is acknowledged that functional remodeling of cortical areas may occur also in glioma patients. Principal limits of previous investigations on cortical plasticity of motor and language functions included scarce reports of longitudinal evaluations and limited sample sizes. This systematic review is aimed at elucidating cortical brain plasticity for motor and language functions, in adult glioma patients, by means of preoperative and intraoperative mapping techniques. We systematically reviewed the literature for prospective studies, assessing cortical plasticity of motor and language functions in low-grade and high-grade gliomas. Eight longitudinal studies investigated cortical plasticity, evaluated by motor and language task-based functional MRI (fMRI), motor navigated transcranial magnetic stimulation (n-TMS), and intraoperative mapping with cortical direct electrocortical stimulation (DES) of language and motor function. Motor function reorganization appeared relatively limited and mostly characterized by intrahemispheric functional changes, including secondary motor cortices. On the other hand, a high level of functional reshaping was found for language function in DES studies. Occurrence of cortical functional reorganization of language function was described focusing on the intrahemispheric recruitment of perilesional areas. However, the association between these functional patterns and recovery of motor and language deficits still remains partially clear. A number of relevant methodological issues possibly affecting the finding generalization emerged, such as the complexity of plasticity outcome measures and the lack of large longitudinal studies. Future studies are required to further confirm these evidences on cortical plasticity in larger samples, combining both functional imaging and intraoperative mapping techniques in longitudinally evaluations.

## 1. Introduction

Cerebral plasticity is the biological dynamic ability of the central nervous system to reorganize itself in response to injuries, such as damages caused by brain tumors [1, 2]. The reorganization of functional areas is a fundamental mechanism to compensate for impaired function [3].

In the last two decades, results from a large body of neuroimaging studies in stroke patients have substantially advanced the current knowledge of brain plasticity mechanisms. Longitudinal neuroimaging studies, in particular functional magnetic resonance imaging (fMRI) reports, point out that language and motor network reorganization changes over time, starting from the postinjury early phase to the chronic phase of stroke [4, 5].

During the acute phase, a damage in critical areas (i.e., left temporal lesion) may lead not only to a local hypoactivation in the lesioned area, but also to a global network dysfunction, which can be worsened by structural white matter fiber tract disconnection [6]. Following the acute phase, functional compensation starts to take place. Indeed in the subacute phase, compensatory mechanisms of upregulation become evident, with an extensive increase of activation of undamaged areas in the lesioned hemisphere and in the intact hemisphere [7]. The compensation role is attributable both to the recruitment of homologous areas in the contralateral hemisphere and to the activations of domain-general areas in the ipsilateral hemisphere (i.e., dorsolateral prefrontal cortex for language and parietofrontal areas for motor function). Major mechanisms of functional reorganization after 6 months comprise the recovery of perilesional brain tissue and spared areas as well as the recruitment of homologous areas in the healthy hemisphere. For language function, the best level of recovery is generally observed when compensatory recruitment takes place in the perilesional area along the rim of the injured tissue together with less activation in the right hemisphere [8]. However, depending on the size and site of the ischemic lesion, recruitment of homologous areas in the right hemisphere seems also to have a supportive role [9]. For motor function, it appears that the continuous overactivation of the contralateral hemisphere is more frequent in patients with partial recovery of symptoms [10].

This cerebral malleability can be achieved also when a tumor is invading an eloquent region and may explain why patients at early stages show mild symptoms [11]. In particular, lower grade infiltrating gliomas are characterized by progressive functional reshaping, due to their slow growth. Such modifications can occur during the natural history of illness or following a treatment [12, 13]. However, the exact mechanisms underlying brain cortical plasticity remain unclear.

A plethora of functional techniques has been exploited to study cortical plasticity and to monitor such functional modifications at different stages of the disease. Among the preoperative techniques proposed to help neurosurgical planning and procedure, functional magnetic resonance imaging (fMRI) represents the most widely used clinical application for mapping functions in the brain [14]. Functional MRI relies on the hemodynamic response related to neuronal activity, which is induced by a stimulus or a task. By measuring blood-level oxygen level dependent (BOLD) signal changes on T2^∗^-weighted images, task-related functional activations can be detected.

Task-based fMRI is now increasingly included in the clinical workup of brain tumor patients in order to provide a noninvasive functional mapping of motor and language areas [15, 16]. Although fMRI is used predominantly as a neuroscience research tool, clinical applications are emerging, especially in the presurgical assessment of motor and language function, in patients with brain tumors, epilepsy, and vascular malformations [14, 17, 18]. Preoperative fMRI provides a localization of eloquent functional cortex and an assessment of the relationship with the tumoral lesion [19]. Notably, fMRI examinations are performed with the final aim of facilitating function-preserving and safe treatment in brain tumor patients, by supporting the identification of specific cerebral functions, such as language [17, 20]. However, so far, the validation of language lateralization based on a task-based fMRI technique is still not conclusive [21–23].

Furthermore, mapping of eloquent cortex can provide useful insights on the mechanisms of neuroplasticity. Not only fMRI is valuable to understand functional brain reorganization longitudinally, but it also allows to simultaneously reveal all the brain regions involved in a task or during rest, differently from other preoperative and intraoperative methods.

Recently, according to the connectome perspective, a novel approach of resting-state fMRI (rs-fMRI) has emerged as a promising tool also in brain tumor [24–26]. rs-fMRI has increasingly contributed to the delineation of intrinsic cortical networks of different domains (sensorimotor, language, and attention) and their characterization in the presence of a tumor. However, the utility of rs-fMRI in presurgical mapping of motor and language function is only partially described, and to our knowledge, no follow-up studies have been performed so far.

Despite of the limited diffusion of magnetoencephalography (MEG) systems, MEG represents another noninvasive technique to aid in the surgical planning by localizing eloquent cortex [27]. MEG estimates the magnetic fields generated by electric currents in the brain, providing a direct measure of brain function, with high temporal resolution. In the clinics, MEG technique is typically used in epilepsy for the localization of the epileptogenic zone and its relation to areas of the eloquent cortex [28, 29]. Since MEG signal is insensitive to the distortive effects of anatomical lesions on brain microvasculature, it can be used in a complementary fashion for presurgical purpose in tumor patients. In particular, in tumor patients with a massive distortion of neuroanatomy, MEG helps to localize the hand motor area and primary sensory area [30, 31]. Preoperative mapping of language-related cortices is also feasible with appropriate tasks (i.e., verb-generation task) [32]. However, the development of MEG applications in presurgical setting is still limited due to the scarcity of MEG systems, as the installation of a MEG device and its service costs are indeed expensive.

In the last years, transcranial magnetic stimulation (TMS) has been increasingly performed in the presurgical setting to localize functional areas, especially the motor cortex [5, 28, 33]. By means of coil-induced transcranial magnetic stimulation of specific cortical brain regions, it is possible to evoke a positive effect similar to the one elicited by intraoperative direct electrocortical cortical stimulation (DES). Hand muscles are activated after stimulating the motor cortex for motor function, while language functions are inhibited after stimulating the perisylvian region, resulting in speech arrest or errors.

Recently, TMS has been successfully integrated with subject-specific brain imaging data, using neuronavigational technology and thus improving the spatial accuracy of the stimulation. This approach is called navigated-TMS (n-TMS), and presurgical motor mapping can be performed by stimulating the rolandic region in patients with tumors and simultaneously recording muscle motor-evoked potentials for each stimulating area.

Remarkably, n-TMS is a reliable and clinically validated tool to identify functional areas belonging to the motor system. Despite increasing evidence is accumulating, the application of n-TMS in presurgical language mapping is still quite limited [31, 34–36].

However, n-TMS can be also useful to delineate cortical plasticity of a motor system in glioma patients, providing a direct measure of cortical response to the induced stimulation.

Although all preoperative mapping techniques represent a unique window to look at functional correlates of a motor and language system in brain tumors, evidences from both fMRI and n-TMS investigations are not univocal. Functional rearrangements, possibly deriving from neuroplasticity, have been examined in different groups of patients with various types of brain tumors, mostly at a single time point before surgery. Moreover, the comparison between brain tumor patients and healthy subjects might be affected by anatomical distortions due to the normalization process.

In order to investigate motor or language cortical plasticity in tumor patients, an appropriate experimental protocol should forecast an intrasubject evaluation of possible functional changes. Nonetheless, it would be arduous to conduct a prospective study following the clinical history of gliomas avoiding the surgical removal, which is the current standard treatment and cannot be denied to any patient.

Optimal candidates for intrasubject examinations of local brain plasticity are patients undergoing repeated awake surgeries, whose brain functions can be mapped intraoperatively by direct electrocortical stimulation (DES) [37, 38]. The DES technique has the advantage to provide a direct measure of the response of a cortical site, even if it can be limited by spatial constraints due to the confined portion of cerebral cortex that can be exposed. Repeated resections in low-grade gliomas (LGG) enable to disclose functional changes in the cortical maps. In particular, cortical sites showing a positive response during the first surgery may be no longer eloquent at the reoperation [39]. Such a functional reshaping can be interpreted in relation to the natural modifications of the perilesional tissue and may reflect plasticity phenomena. This aspect is particularly useful to optimize the extent of resection in traditional “critical” areas, such as the primary motor cortex, premotor region, and superior temporal areas, as demonstrated in a number of studies [13, 40, 41].

Neuropsychological assessment is another fundamental measure of the functional outcome related to plasticity. It evaluates the cognitive function of patients suffering from glioma and allows to monitor the progression of deficits over time [42–44]. Furthermore, an objective measure of cognitive deficits is crucial to understand the association between functionality loss or recovery and brain plastic adaptations, in particular for slow-growing lesions.

Previous studies have not fully addressed the dynamic process of neuroplasticity in glioma patients, thus a number of open issues remain. First of all, it has not been established which is the most appropriate mapping technique or the possible combination of different mapping methods to identify plasticity-related changes. Secondly, variable findings have emerged regarding the reorganization patterns of cortical motor and language areas, as well as the measures to assess these functional changes. Moreover, it has not been defined a specific timing for performing functional examinations or the correspondence between a precise time point of evaluation with a given pattern of rearrangement. Lastly, it is not clear how neuropsychological measures may reflect the clinical significance of these functional reorganization processes.

The present systematic review of the literature is aimed at examining studies that assess brain plasticity of motor and language functions, during the clinical history of brain gliomas, using preoperative functional neuroimaging and intraoperative techniques.

## 2. Methods

### 2.1. Search Strategy

Electronic database PubMed was systematically reviewed for literature published between January 2000 and September 2018. The search strategy consisted of a combination of two search strings: those related to primary brain tumor and those related to plasticity. The full search string used was as follows: *Brain Tumors OR Glioma OR Epilepsy brain tumor* AND *Neuroplasticity OR Language Plasticity OR Sensorimotor Plasticity OR Motor Plasticity OR Plasticity fMRI OR Plasticity Cortical Stimulation OR Plasticity Transcranial Magnetic Stimulation OR Plasticity Magnetoencephalography OR Plasticity Resection OR Plasticity Neuropsychology OR Neuroplasticity Neuropsychology*.

### 2.2. Selection Strategy

**Figure 1:**
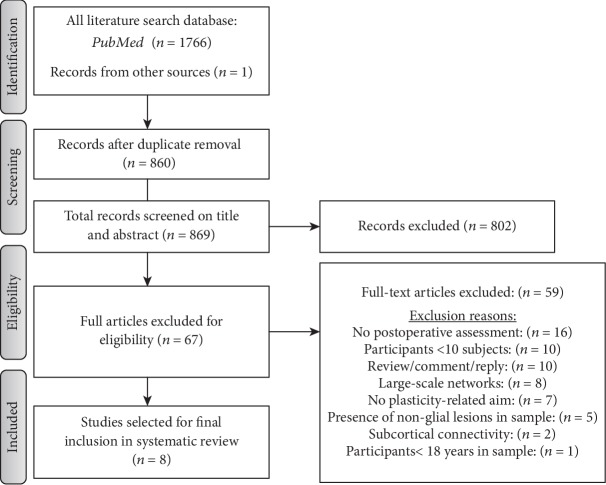
PRISMA flow diagram of the conducted systematic search.

The flow diagram of the Preferred Reporting Items for Systematic Reviews and Meta-Analyses (PRISMA) is provided in Figure 1.

As shown in the flow diagram, the first search resulted in 1766 records. After removal of the duplicates, 869 articles were screened based on title and abstract.

In order to be included records had to fit the following inclusion criteria: (i) glioma tumor patients, (ii) ≥10 patients studied, (iii) adult population (>18 years), (iv) language or motor function cortical plasticity, (v) presurgical examinations performed with fMRI or TMS or MEG *and* postsurgical examinations performed with the same mapping technique *and/or* postsurgical clinical/neuropsychological evaluation, and (vi) intraoperative assessment performed with awake DES combined with clinical and/or neuropsychological assessment. The exclusion criteria were as follows: reviews, meta-analyses, comments, or replies.

It was determined that 802 of these studies did not meet the inclusion criteria. Sixty-seven records were selected for further detailed screening, examining the full-text article. Fifty-nine articles did not fit the inclusion criteria, as described in detail in Figure 1. In total, 8 articles met all the inclusion criteria and were identified for the review.

## 3. Results

**Figure 2:**
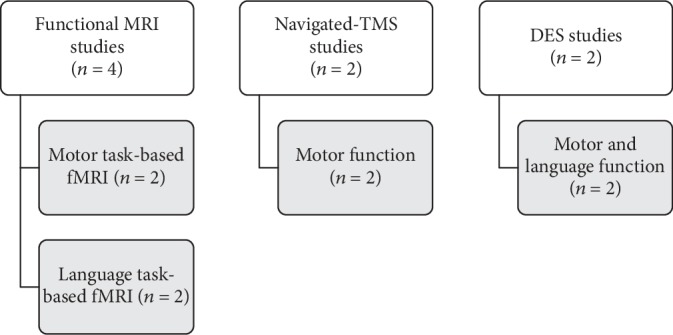
Summary of the included studies depending on the mapping technique.

Eight prospective studies investigated motor or language cortical plasticity evaluated by motor [45, 46] and language [47] task-based fMRI, motor [48, 49] navigated-TMS, and intraoperative evaluation with cortical DES of language and motor function [50, 51], as shown in Figure 2.

During the selection process, a number of studies were excluded due to multiple reasons: small number of participants included (<10), absence of an instrumental postsurgical assessment, nonmotor or nonlanguage functional reorganization (i.e., large-scale or attentional networks), and reviews or commentaries. Detailed description is provided in Figure 1.

### 3.1. Participants' Characteristics

The total number of patients that were studied was 170. The sample size ranged from 10 [52] to 42 participants [50]. Patients' mean age ranged from 30.9 ± 7.4 years [50] to 53.1 ± 13.1 years [46]. Only one paper reported the participants' level of education [47].

Studies regarding motor reorganization included patients with tumor located in primary and secondary motor areas (M1, supplementary area, and premotor area) in both hemispheres, while studies regarding language reorganization included patients with tumors located predominantly in the left hemisphere in the frontal and temporal language areas.

Tumor size was specified in five studies (5/8) [45, 46, 50–52], one reported only resected volume [47]. The extent of resection was included in six investigations (6/8) [45, 46, 48–51]. Radiological tumor features, tumor remnant size, and volume of postoperative cavity were described only in one study [50].

Except for one n-TMS paper [48], which included three patients (3/20) with previous surgery, the remaining seven investigations examined patients without previous surgical treatment. Intraoperative stimulation studies included patients who underwent two or more surgeries. In detail, one study compared two surgical interventions [50]; in the other one, 2 patients (2/20) underwent more than two surgical removals [51].

Information about progression of disease, epilepsy, pharmacological treatment, chemotherapy, and radiotherapy of patients was sparsely reported.

All the studies applied a within-subject experimental design, consisting in the comparison of preoperative *vs*. postoperative fMRI or n-TMS examinations. Two articles added a between-subjects analysis, comparing patients *vs*. healthy controls [47, 48] and left-side tumor patients *vs*. right-side tumor patients [47].

The interval between preoperative and postoperative examinations was variable depending on the mapping technique. Functional MRI examinations ranged from 1 month before to about 4 months after surgery (maximum 126 days). Motor n-TMS were highly variable regarding their timing. Conway et al. [49] performed the n-TMS within 1 year, whereas Barz et al. [48] included patients studied with a mean preoperative time of about 2 years (mean months pre: 26.1 ± 24.8) and after until about 4 years (mean months post: 46.3 ± 25.4). Repeated intraoperative surgeries were also performed with a time interval within 4 to 5 years.

A summary of the articles included in the review is provided in Table 1.

### 3.2. Plasticity Outcome Measure

**Figure 3:**
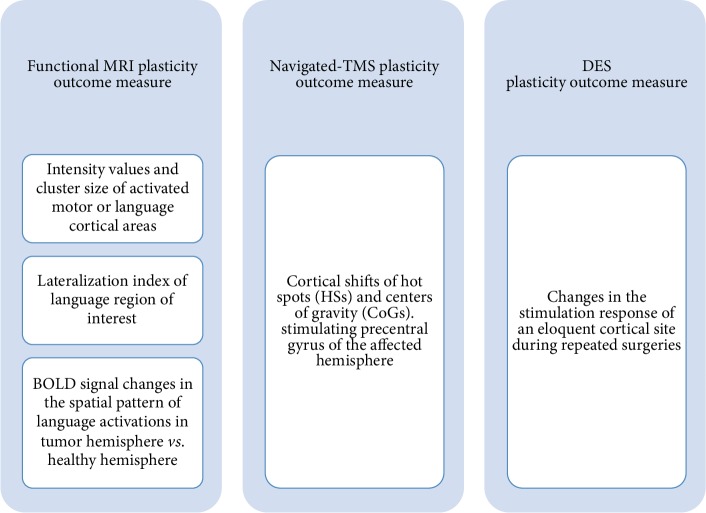
Description of plasticity outcome measures.

As expected, each preoperative and intraoperative mapping technique adopted different measures to assess functional reorganization of motor and language systems. A schematic representation on specific measures adopted from the studies is given in Figure 3.

Motor fMRI studies were comparable in terms of plasticity outcome variable, as well as n-TMS articles. Notably, language fMRI studies considered different plasticity outcome measures. It is worth highlighting that fMRI could provide a whole assessment of the spatial pattern of functional activations, both in the hemisphere affected by the tumor and in the contralateral hemisphere. In contrast, DES mapping and n-TMS were able to provide information about the local reorganization. While DES is confined to the cortical surface exposed during surgery, n-TMS is routinely exploited to specifically perform stimulations focused on areas of interest and, despite the possibility of performing multiple stimulations, it is most commonly targeted to one single area.

Regarding motor plasticity, fMRI investigations considered the changes, for primary and secondary motor areas, in the intensity values (a statistical score of each activated area) and in the cluster size (the dimension of each activated area), as a plasticity outcome measure [45, 46].

Motor n-TMS studies analyzed the changes of cortical representations of hand and leg areas in the motor cortex of the affected hemisphere, measuring the hot spot (HS) and center of gravity (CoG) shifts [48, 49]. In particular, HS consisted in the cortical point in which the stimulation elicited the largest motor-evoked potential (MEP), upon a specified intensity of stimulation. Center of gravity (CoG) of the MEPs corresponded to a weighted mean of the stimulations' amplitudes of the corresponding 3D spatial coordinates of a valid stimulation point, for a number of trials. For motor reorganization, intraoperative mapping studies adopted a measure based on the response to the stimulation of an eloquent cortical site. The stimulation of a motor eloquent site can elicit a positive response (i.e., hand movement), indicating the real eloquence of that cortical site or a negative response (i.e., absence of movement). Changes in the response to the cortical stimulation of two overlapping sites were used to define if a cortical site remained stable or if it was lost or gained from the first to the second surgery. Precise alignment of hand-drawn maps and digital photographs of numerical cortical markers were attentively observed to compare mapping results during surgeries. Both Picart et al. [50] and Southwell et al. [51] described the changes in the stimulation responses for motor cortical sites across more than one surgery.

On the other hand, regarding language plasticity, Kristo et al. [47] introduced a method to quantify the difference, at two fMRI examinations, in the spatial distribution of language activations in both hemispheres. They precisely defined functional reorganization as the mean differences in BOLD signal before and after surgery, discriminating changes in the spatial pattern of the BOLD signal from changes related to the whole brain BOLD signal. The difference was computed between the two scanning sessions, taking into account cortical regions both in the affected and in the healthy hemisphere for each patient. Functional MRI signal can vary due to global brain variations, affecting the amplitude of BOLD responses to a similar extent across the entire brain. This type of variation left the spatial pattern of activation relatively unchanged. Nonetheless, the underlying signal could also differ due to variations in the spatial pattern of activation related to specific functional modifications. This pattern is defined as a standard deviation from the global signal. At two fMRI examinations, the variation of the distribution of spatial pattern of BOLD signal (true variation) was distinguished from those variations related to a global brain effect (noise), and this difference was computed for every cortical area. Through this quantitative method of analysis, the authors were able to precisely estimate spatial functional variations which occurred across time in the healthy hemisphere.

In contrast, in the other fMRI study, Gebska-Kosla et al. [52] observed, for each patient included in the study, language activation changes in the intensity and in the cluster size of ipsilateral and contralateral Broca's and Wernicke's areas, despite a lack of group analysis of the patients' sample. Changes in the lateralization index (LI) of language areas were also considered. LI was defined as the result from (L-R)/(L+R) and ranged from -1 (right lateralized) to 1 (left lateralized).

As mentioned for language plasticity, DES studies used a measure based on the response to the stimulation of an eloquent cortical site. The stimulation of a language eloquent site could induce a transient deficit (i.e., speech arrest or anomia), indicating the real eloquence of that cortical area, thus defined as a positive site. The absence of a deficit subsequent to the stimulation, in contrast, indicates the noneloquence of that site, thus defined as “negative.” As for motor mapping, DES studies evaluated the changes in the response to the cortical stimulation of two overlapping sites in order to define if a cortical site remained stable or if it was lost or gained during the time interval between surgeries. Both Picart et al. [50] and Southwell et al. [51] described the changes in the stimulation responses for language cortical sites across more than one surgery.

### 3.3. Motor, Language, and Cognitive Assessment

The Lovett scale (Lo) was performed to assess the degree of upper limb paralysis, by evaluating muscle strength. It was used in two motor fMRI investigations [45, 46]. In the remaining n-TMS and DES studies, no information about the testing instrument was provided and a general indication on the presence/absence of preoperative motor deficit and postoperative progression of symptoms was given.

Other authors did not specify which language test or battery was administered, and a general information (i.e., mild aphasia) on patients' language deficit and its severity was included in the study [50–52]. In neuropsychological assessments, which were reported in two fMRI articles [45, 47], the following cognitive domains were evaluated: intelligence level, abstract reasoning, attention, working memory, and visuomotor coordination.

### 3.4. Motor Function Reorganization: Evidence from DES, n-TMS, and fMRI Investigations

Picart et al. and Southwell et al. tried to identify cortical plasticity during repeated awake surgeries in patients suffering from both low- and high-grade gliomas, located in different eloquent brain areas [50, 51]. They evaluated cortical mapping results at repeated surgeries.

Picart et al. [50] classified eloquent cortical sites as stable during the second surgery (positive-positive or negative-negative response), lost (positive to negative response), or gained (negative to positive response) in comparison to initial surgery. Displacement of more than 2 cortical sites exposed twice was interpreted as a sign of plasticity, and it determined if a patient was considered with a “high level” of cortical plasticity (group 1: remapping) or a “low level” of plasticity (group 2: without remapping).

In the remapping group (23 patients), 20 motor sites were detected during the first mapping and 35 motor sites were identified during the second mapping. In the nonremapping group (19 patients), 35 motor sites were detected during the first mapping and 36 motor sites were identified during the second mapping. Comparing the two groups of patients, in the remapping group, the rate of stable motor eloquent sites was significantly lower than in the nonremapping group (65% vs. 97.1%). Moreover, the rate of gained motor sites at reoperation was higher for the remapping group compared to the nonremapping group. In particular, the primary motor area was displaced in 4 patients. In a patient presenting a diffuse glioma involving the left precentral gyrus and posterior part of the middle frontal gyrus, at the second surgery in comparison to the first one, the primary motor area was found more posteriorly. Clinical, demographical, and tumor characteristics did not differ significantly between the two groups, except for the radiological pattern. In remapping patients, there was a higher rate of tumors with sharp borders compared to the nonremapping group, in which tumors presented indistinct borders.

Southwell et al. [51] examined 18 patients who underwent repeated awake surgeries in which one or more cortical eloquent sites had been tested in the initial surgery. More than half (66.7%) of the 9 motor sites were identified as stable (positive-positive response), while a percentage of 33.3% of motor sites exhibited a loss of function (positive-negative) in response to the stimulation across two surgeries. None motor cortical sites displayed a gain of function. Furthermore, in those patients who displayed a change in DES (7/18), only 2 out of 7 patients showed a modification in the stimulation results for motor function from positive to negative response. These patients presented low-grade gliomas located in the frontal region. However, this loss of motor function was not associated with the onset of new motor deficits.

Two recent works, using navigated transcranial magnetic stimulation (n-TMS), described the shape of functional changes in the cortical motor function, for hand and leg areas, in perirolandic tumors of different grades.

Conway et al. [49] compared hot spot (HS) and center of gravity (CoG) shifts of cortical representations between posteroanterior and mediolateral direction, at two time points. For both HSs and CoGs, a greater significant shift along the posteroanterior direction was found compared to the mediolateral shift. Even if not statistically significant, lesions located more anteriorly (near premotor areas) shifted toward an anterior direction, while posterior tumors (near the postcentral gyrus) displaced toward a posterior direction. Shifts also positively correlated with time interval between pre- and postoperative TMS examinations, despite the fact that no correlation between the motor deficit severity and HS and CoG shift changes was performed.

In the second study, Barz et al. [48] did not find any statistically significant group difference in patients. Only in three patients, a significant difference of CoG shifts along the anteriorposterior direction was identified during a postoperative period. Remarkably, none of them had tumor progression or radiotherapy and 2 of them had a full recovery of motor deficits. No association of motor cortex reorganization with tumor size or extent of resection was found.

Two fMRI investigations, from the same group, evaluated motor function reorganization, comparing the preoperative and postoperative intensity and the extent of cluster of activation of primary and secondary motor cortex in low-grade gliomas (LGG) and high-grade gliomas (HGG) [45, 46]. During the fMRI study, patients had to perform a simple motor task with the upper limb contralateral to the brain lesion.

Bryszewski et al. [45] recruited 20 patients with a low-grade tumor located within the motor or sensory cortex. A pattern of motor functional reorganization characterized by the activation of bilateral motor primary cortices, premotor areas (PMAs), and ipsilateral SMA was identified both before and after surgery. Although statistically not significant, three months after surgery, these motor areas tended to increase their intensity of activation and tended to decrease their cluster size, except for ipsilateral PMA.

Clinically, more than one-half of patients did not present motor impairment, and the remaining had only slight deficits. Postsurgery, half of the patients remained free from motor deficits. Thus, no postoperative group difference for the motor impairment was found. Furthermore, after surgery, comparing patients with a slight motor deficit with nonimpaired patients, the former presented significantly higher intensity values of ipsilateral SMA.

Majos et al. [46] observed a similar pattern of motor-activated areas in 16 highly malignant tumors located within the motor and sensory cortex. However, different from lower-grade malignant tumors, in HGG, a postoperative trend of decrease in the intensity as well as in the cluster size of activation of primary motor area and premotor area, both in ipsilateral and contralateral hemispheres, was found. The only area which remained stable across the two exams was the ipsilateral SMA. Preoperatively, patients presented moderate motor deficits, which worsened three months after surgery.

### 3.5. Language Function Reorganization: Evidence from DES and fMRI Investigations

Regarding DES studies on language reorganization, Picart et al. [50] found that in the remapping group (23 patients), 82 and 56 language cortical sites were detected, respectively, during the first mapping and the second mapping. In the nonremapping group (19 patients), 36 and 40 language cortical sites were identified, respectively, during the first mapping and the second mapping.

At the second surgery, the majority (83.3%) of language eloquent cortical sites was stable in the nonremapping group. In contrast, the remapping group showed a higher level of reshaping since a lower percentage (23.2%) of eloquent language sites was found as stable. Language cortical function exhibited a higher level of reshaping, particularly in the dorsolateral prefrontal cortex in 13 patients and in the ventral premotor cortex in other 13 patients. The rate of lost language sites at reoperation was higher for the remapping group compared to the nonremapping group (76.8% *vs*. 16.7%).

Southwell et al. [51] identified that 6 cortical sites of the 101 tested for language exhibited a loss of function during repeated surgeries (positive-negative response), while only 1 language site showed a gain of function (negative-positive response). The majority of language sites demonstrated a stable negative response to DES. However, in those patients who demonstrated a change in DES (7/18), the majority of them showed a functional modification in the stimulation response for language function. Four patients displayed a positive-negative change, while one patient showed a negative-positive change. They had low-grade gliomas located in the frontal, insular, and temporal regions. Remarkably, loss of function was not associated with the onset of new language deficits at the time of the repeated surgery.

Kristo et al. [47] used a quantitative novel technique to discriminate between “noising” activation changes due to whole brain effects and “true” changes in the spatial pattern of activations, performing a group analysis in LGG patients [47]. Functional MRI was performed using a verb-generation task (with visual stimuli). Mild language deficit was present in few patients and cognitive performance was on average, both preoperatively and postoperatively.

The authors focused on language-task-induced activations in the right healthy hemisphere before and after resection. Both preoperatively and postoperatively patients with left gliomas showed higher activation of the left hemisphere compared to the right one. Notably, five months after surgery, the greatest modifications in spatial patterns of activations were close to the surgical resection in the left hemisphere. Furthermore, no difference was found between patients with recovery of language impairments compared to patients without functional recovery for the spatial pattern of activations.

Gebska-Kosla et al. [52] described functional language rearrangements in HGG and LGG patients. Functional MRI was performed using a word-generation task (with visual stimuli). They evaluated in each single patient language intensity activations, cluster size, and lateralization index for two regions of interest, Broca's and Wernicke's areas. During the postoperative period, they found a heterogeneous pattern of language reorganization, associated with language impairments. In the group of frontal LGG (5 patients), the right-side Broca's area was activated in patients without speech disorders, both preoperatively and postoperatively. As far as the language impairments are concerned, one frontal LGG patient, who presented transient motor aphasia, remained stable after surgery and presented a decrease in left Broca's activation, with a left-side dominance. In another case of frontal LGG, the increase of left Broca's activation and the appearance of right Broca's activation were associated with the recovery of aphasic symptoms, with a stable left-side dominance. In the group of temporal HGG (5 patients), the right-sided Wernicke's was activated in all patients, both preoperatively and postoperatively. Worsening of language disorder was associated with the lack of functional activation of both left and right Wernicke's in one temporal HGG patient.

## 4. Discussion

This systematic review examined how plastic rearrangements of cortical topography can occur in the natural clinical history of gliomas, for motor and language systems, as revealed by means of preoperative and intraoperative mapping techniques.

We identified eight articles in which the functional cortical reorganization was investigated. Overall, both intra- and interhemispheric modifications were reported in the short and long term, indicating that cortical reorganization processes may occur in patients. Nevertheless, not all the reviewed studies that exploited different mapping techniques univocally claimed conclusive evidence on motor and language plasticity in gliomas.

Eventually, useful suggestions will be provided with the aim to possibly harmonize future studies focusing on cortical plasticity in glioma patients.

### 4.1. Methodological Issues

Despite many claims on functional reorganization, empirical studies that systematically investigated cortical plasticity in glioma patients are extremely limited. Indeed, most of the studies investigated neuroplasticity at one-time point only, for instance, presurgically. The lack of prospective studies, especially using rs-fMRI and MEG, explains why only eight works have been included in the present review.

First of all, it emerged that a general consensus on the definition of cortical plasticity is still far from being uniform. In particular, it has not been explicitly defined a single outcome variable that could measure specific changes related to brain functional reorganization.

As disclosed by the reviewed language fMRI studies, divergent methods adopted to measure language plasticity have prevented the gathering of comparable results, even if the same preoperative mapping procedure has been used. In the fMRI technique, the comparison based on the intensity values and the cluster size of functional activations can be confounding, given that the subject's task performances and cognitive efforts are hard to be controlled, especially in patients with language impairments. The novel method proposed by Kristo et al. [47] seemed to discriminate more precisely whether activation modifications reflect changes related to global effects in BOLD signal amplitude or changes in the spatial pattern distribution of BOLD signal. This difference is fundamental to avoid ambiguity in the interpretation of the nature of fMRI activations in patients [53]. Functional changes related to global effects can be determined by differences in individual task performances or in anatomical misalignments between scanning sessions. In contrast, changes in the distribution of a spatial pattern of functional activations are thought to reflect mostly functional reorganization. Furthermore, individual intrasubject functional activation changes can be very subtle in terms of BOLD signal variation. Therefore, a robust outcome variable along with a group analysis might be more suitable for the identification of such small signal modifications in fMRI activations. Additionally, it is acknowledged that fMRI activation maps in the same subject can contain substantial variation across sessions [53]. The noise is produced both by the scanner and by human physiological processes such as heartbeat and respiration. When studying cortical plasticity, an ideal prospective protocol in tumor patients should forecast an intrasubject evaluation of plasticity measure at different time points. Furthermore, the same protocol should be performed also in healthy-matched individuals (single and\or control group) in order to account for normal variations depending on the method. The comparison with a normative sample would allow to highlight more efficiently the true signal variation that actually reflects functional changes induced by plasticity, by distinguishing it from the background noise. This methodological aspect is fundamental to appreciate individual changes in a functional network.

None of the reviewed fMRI investigations applied advanced functional imaging analysis methods, such as dynamic casual modeling, which is performed to characterize effective connectivity within cortical networks. This is quite surprising because the application of a complex model of signal analysis can potentially improve the possibility to detect subtle variations in highly dynamic networks.

Another limit is represented by the discrepancy in language fMRI paradigms used in the studies. In Kristo et al., a verb-generation task was performed, whereas Gebska-Kosla et al. applied a word-generation paradigm [47, 52]. Given that conclusions about the patterns of language reorganization are based on findings derived from a single linguistic task, an important limit in studying cortical plasticity is constituted by the lack of standards in language fMRI paradigms.

Regarding n-TMS plasticity outcome measure, Conway et al. [49] adopted both hot spot (HS) and center of gravity (CoG) shifts of motor cortical representations, while Barz et al. [48] considered only CoGs. CoG may be a more reliable variable compared to HS measure because, being a weighted measure of a number of trials, it is less affected by differences in coil orientation. However, multiple stimulations are needed to calculate a valid CoG, which it may be challenging in the clinical setting.

Another crucial limit is the small size of samples included in most of the investigations that may prevent from finding significant modifications in patients, due to poor statistical power. The majority of the reviewed fMRI studies [45, 46, 52] failed to identify statistically significant differences in motor and language activations just because only few patients have been studied by means of two fMRI examinations. In the literature, overall, the majority of studies described cortical plasticity only in small patients' samples or single cases [12, 54], but substantial evidence derived from the analysis of more consistent samples is still lacking. Recruitment of a large and homogenous sample of glioma patients in a prospective protocol can be challenging, due to the large heterogeneity of patients' characteristics and tumor-related features, i.e., age at the diagnosis, clinical and cognitive impairments, tumor location, tumor grade, and clinical treatment.

Ultimately, a consideration regarding the timing of examinations is necessary. On the one hand, fMRI has been performed at different time points with respect to DES, but those two groups are internally homogeneous. In fact, fMRI is a short-term evaluation performed 3-4 months after surgery, while DES studies long-term brain modifications at 4-5 years. On the other hand, the time intervals considered in the group of n-TMS evaluations were greatly variable, making this group the hardest to be analyzed.

The heterogeneity in measures for an objective quantification of cortical plasticity represents an important concern, as it limits the comparison between studies and thus the possibility of determining convergent evidences about functional reorganization mechanisms.

### 4.2. Motor Function Reorganization Processes

Despite of different pathophysiology occurring between stroke and glioma, well-established cortical plasticity models of stroke may help to better understand functional rearrangements shown in tumor lesions.

Ischemic strokes are caused by an interruption of blood supply due to an obstructed blood vessel. Consequently, brain tissue in the affected vascular territories becomes dysfunctional and ultimately necrotic. After the hyperacute and acute phase, the reperfusion of the ischemic penumbra contributes to clinical improvements in stroke patients. However, the acute feature of cortical brain injury together with subcortical injury may account for permanent deficits observed in stroke patients.

Different from the acute onset of stroke, lower grade gliomas are infiltrating lesions, characterized by a slow growth. Progressive lesions might be associated with a higher potential of long-term recovery, together with a redistribution of function within the network, except in the case of extremely large cortical damage.

As mentioned, neuroimaging findings in stroke studies shed light on major mechanisms involved in cortical neuroplasticity, such as the cortical recruitment of perilesional synapses or homologous areas.

**Figure 4:**
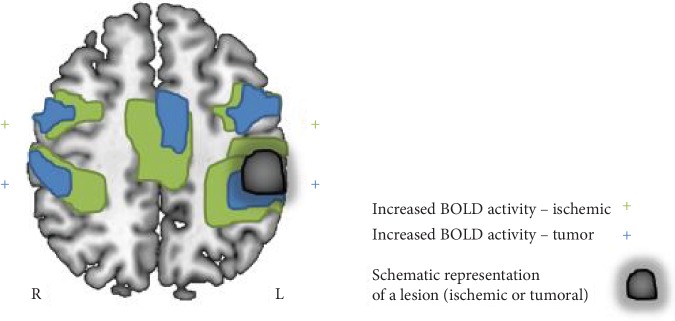
Schematic representation of the major mechanisms of functional reorganization of motor function in the subacute phase of a stroke lesion and postoperatively in a tumor lesion. Functional MRI activations of primary and secondary motor areas during the subacute phase of a stroke lesion (green color) and postoperatively in a low-grade glioma (LGG) (blue color) in the left precentral region. Comparison of two models of reorganization shows a similar pattern of motor-activated areas, but an overactivation of ipsilesional and contralesional areas (M1, premotor area, and supplementary motor area) is evident in ischemic lesion compared to LGG.

Regarding motor function plasticity, stroke patients, during the subacute phase, 3 to 6 months poststroke, typically experienced an overactivation of primary and secondary motor areas of both lesioned and intact hemisphere (Figure 4). Redundant synapses are normally inhibited by interneurons, but when an injury occurs, this inhibition from the lesioned to the contralateral hemisphere, via transcallosal fibers, is reduced. Transcallosal fibers may be used to integrate areas from the healthy hemisphere in the neuronal computations necessary for movement planning and execution. Successful motor recovery in these patients is associated with a normalization of overactivation, resulting in a restored interhemispheric balance between lesioned and healthy hemisphere motor activation. Moreover, normalization of activity in ipsilesional primary motor and premotor areas in chronic patients signals good motor recovery.

Comprehensively, fMRI investigations showed that a relative short-term rearrangement may occur, involving mostly secondary motor areas rather than primary motor areas. Secondary motor areas, as premotor and supplementary cortex, seem to play an auxiliary role when motor worsening arises.

In particular, three months after surgery, the recruitment of secondary motor areas in both hemispheres and contralateral primary motor cortex appears to be one of the possibly reorganization mechanisms for motor function (Figure 4).

This kind of rearrangement is already evident in LGG patients with mild motor impairment, as showed in Bryszewski et al. study. Notably, LGG patients did not show clinical and cognitive impairments both pre- and postoperatively, suggesting that this rearrangement process may be efficient. Furthermore, postoperatively, in LGG patients, the ascending trend of increased intensity of activated motor areas, mostly ipsilateral PMA and SMA, could be interpreted as an adaptive marker of an initial recovery of a motor system. Even if not statistically significant, the decrease observed in a cluster size of activated motor areas of LGG patients possibly reflect a more precise defining of motor center activations after surgery. In healthy subjects, it was described that functional activations of M1 were more homogenous and precisely located in the precentral gyrus and that both mean intensity values and cluster size were lower than in patients with proliferative lesions [55].

In HGG, a descending trend of the intensity and cluster size of bilateral motor areas, except for SMA, emerged three months after surgery and it was associated with the worsening of motor deficit. A possibly explanation of the observed hypoactivation of motor areas may be related to tumor effects on cerebral vasculature. Recent studies have demonstrated that mostly high malignant proliferative lesions can exhibit neurovascular uncoupling, which can confound the interpretation of fMRI data [56, 57]. Lesion-induced neurovascular uncoupling indeed induces a decrease of fMRI signal in the perilesional eloquent cortex. On the other hand, the stability of ipsilateral SMA activation before and after surgery was consistent with previous findings [58, 59], suggesting that the SMA may assume a pivotal role in the execution of primary motor activities, mostly in patients with HGG invading the primary motor cortex.

Motor function reorganization, disclosed by the fMRI technique, appeared relatively limited and predominantly characterized by compensatory involvement of secondary motor cortices mainly including intrahemispheric functional changes.

In a similar way, motor n-TMS studies pointed out a cortical reorganization characterized by intrahemispheric rearrangement of cortical motor representations. Such a local reorganization encompassed regions partially belonging to secondary motor cortices, as demonstrated by the direction in anteroposterior axis of HS and CoG shifts. Nonetheless, some concerns need further explanations. In Conway et al. [49], half of patients showed a shift greater than 10 mm at the cortical surface level in HSs and CoGs, which is considered a cut-off ascribable to neuronavigational inaccuracy. This is a critical point determining the accuracy of method, possibly depending on errors and distortions induced by the normalization step. On the other hand, if it would be the case, also shifts in the mediolateral direction would have been affected too. Both n-TMS studies might have been limited by the larger time interval variability between the preoperative and postoperative examinations, some in the order of months and some others of years. Therefore, it is arduous to discriminate whether short-term or long-term plasticity has been investigated in these studies.

Limited, even if present, plastic potential of motor cortices was also confirmed by results from intraoperative stimulations. Interestingly, recently published atlases of functional plasticity and cortical resectability characterized primary motor cortex as a region with limited, even if present up to a certain level, plasticity plastic potential [60, 61]. Both DES studies observed in low-grade tumors that more than half (65% and 66.7%) of motor eloquent cortical sites displayed a stability in the stimulation response across different surgeries. However, in patients who exhibited a change in DES response, after years from the first surgery, motor eloquent cortical sites remapped in areas including the perilesional cortical tissue.

Taken together, these studies on a motor system showed only partial evidence of functional cortical reorganization, further confirming a low potential of plasticity in the primary motor cortex.

### 4.3. Language Function Reorganization Processes

**Figure 5:**
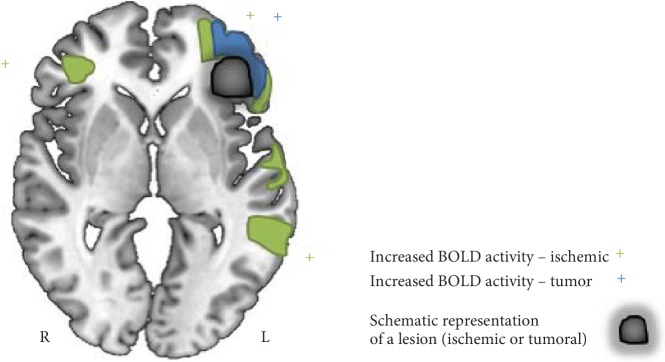
Schematic representation of the major mechanisms of functional reorganization of and language function in the subacute phase of a stroke lesion and postoperatively in a tumor lesion. Language fMRI activations in a stroke lesion (green color) and in a LGG (blue color) in the left inferior frontal region. Notably, perilesional activations in the left inferior frontal regions along the lesional rim are found both in stroke and in LGG, while additional compensatory activations, i.e., in the right homologue areas are more frequently observed in ischemic lesion.

Major recovery mechanisms at play in language recovery are reperfusion of affected areas and recovery from diaschisis, which consists in a dysfunction in a brain region due to loss of long-range inputs from lesioned areas. In general, reperfusion is responsible for early recovery of language deficits, whereas neuroplastic reorganization of language function is thought to regulate more gradual recovery [62]. In the subacute phase of stroke, language reorganization included compensatory mechanisms of upregulation resulting in an extensive increase of activation of ipsilesional spared areas and homologous areas in the contralesional hemisphere, such as right frontal regions (Figure 5). In particular, it was postulated that the perilesional activation tends to increase over time and continues during the chronic phase. A supportive role of domain-general systems, such as those implicated in cognitive control, was also reported, particularly for left temporal stroke [63]. Saur et al. [7] postulated that in temporal lesions, early network disruption seemed more pronounced compared to frontal ones and that lesion-homologue recruitment occurred less than in frontal stroke.

Few studies have systematically investigated cortical plasticity in large samples during the clinical course of the glioma using the fMRI technique. Indeed, only one fMRI investigation on language reorganization in frontal gliomas has been reported. Here, Kristo et al. [47] suggested that five months after surgery, the largest functional changes found in language activations occurred in the proximity of the surgical resection cavity, compared to the homologous areas in the right hemisphere (Figure 5). Although it cannot be excluded that these changes were a consequence of surgery treatment in those areas, the activation of language areas in the proximity of tumor lesion may account for cortical plasticity. In any case, it is of particular interest for the methodology used to discriminate the nature of language fMRI activations in patients and also for the accurate experimental setting (i.e., sample size and language and neuropsychological assessment).

Contrariwise, findings from Gebska-Kosla et al. [52] have to be considered an attempt to identify language plasticity in LGG and HGG patients. Reorganization pattern appeared to be present both in frontal and in temporal gliomas of different grade, and it was characterized by interhemispheric activations in the homologous language areas. However, the complexity of language system makes it difficult to delineate at which extent homologous areas in the nondominant hemisphere can take over for language compensation, when frontal or temporal language nodes are lesioned as well as when a language impairment occurred.

Variability in the language activation patterns both in frontal and temporal patients made it difficult to draw straightforward conclusions. As mentioned before, findings derived from single cases were undoubtedly of interest, but they suffered from limited generalization and were not included in this review. Stronger evidence seemed to arise from fMRI studies focusing on presurgical plasticity [64, 65]. Wang et al. studied presurgical mapping of language areas in a numerous group of 43 patients with a picture-naming task. Patients with left temporal gliomas showed a decrease of activation in Wernicke's area without the activation of the homologous area, while patients with left frontal gliomas displayed compensatory activation of homologous area in the right hemisphere [64].

Despite suggestive evidences of cortical language rearrangements, mostly with intrahemispheric pattern of perilesional areas, the association between functional patterns and language deficit recovery in glioma patients has to be further clarified.

Regarding intraoperative mapping, DES studies demonstrated that language function is characterized by a high level of plastic reorganization, compared to motor and sensory systems. Functional reshaping of language function was found mostly in the dorsolateral prefrontal cortex and ventral premotor cortex of the affected hemisphere. More importantly, loss of function was not associated with the presence of new language deficits, suggesting that these plastic processes were adaptive. Rearrangement processes occurred at long term since the time interval between two surgical treatments was about 4 to 5 years. The long interval time between two surgical interventions could have facilitated an efficient process of functional rearrangement. Although the DES technique may not be able to fully identify the spatial distribution of remapped language areas, due to its intrinsic spatial constraints, it may play a pivotal role in patients undergoing repeated surgical removals. This suggests that time plays a fundamental role in the dynamic process of neuroplasticity mechanisms.

Preliminary evidence on how appropriate stimulation of peritumoral residual can accelerate plasticity changes after first surgical treatment has been provided by a recent report. The authors have showed that the suppression of the eloquent areas within the tumor by continuous cortical electrical stimulation, coupled with appropriate behavioral training, can induce plastic reorganization after less than a month in the contralateral hemisphere. Moreover, this “prehabilitation” has allowed for a more radical extension of resection in the second surgery, since tumoral areas have been no longer eloquent. Although only five patients have been studied and the invasiveness of such experimental protocol, these finding are of great interest as they highlight the extreme plasticity potential of the brain in glioma patients.

### 4.4. Neuropsychological Outcome

Cognitive preservation is essential in glioma surgery, since it is a relevant aspect of daily life functioning. Neuropsychological testing can be useful to determine the cognitive efficiency prior to any treatment and after it, in order to follow the functional recovery and to document the effect of interventions. Cognitive function has an independent prognostic value in glioma patients, as do age, clinical status, and histology [66]. In addition, cognitive deterioration may help to identify tumor progression before signs of disease recurrence are evident on computed tomography (CT) or MRI examination [67]. A recent literature review on neuropsychological testing in glioma patients pointed out that cognitive evaluation has to include all cognitive domains and it should follow a precise time schedule [43].

Despite being stated as essential to understand patients' cognitive functioning, detailed neuropsychological evaluation and specific language assessment using a defined protocol are not always performed in fMRI or n-TMS investigations [68].

This issue characterized also the studies included in this review. Conversely, DES studies performed in institutions with a consolidated experience of awake surgery included cognitive assessment in the standard protocol of glioma patients [69]. In addition, single tests were administered instead of a complete battery for cognitive function evaluation. Further investigations might elucidate which neuropsychological instruments are more sensitive and specific for tumor population in order to identify not only severe deficits but also slight cognitive impairments in patients.

Functional MRI investigations did not provide sufficient evidence of a robust association between patterns of language function reorganization and language and cognitive outcomes. However, DES studies demonstrated that cortical reshaping of language eloquent sites is not associated with the occurrence of new deficits in patients undergoing repeated surgeries. Future studies are needed to investigate more deeply the relationship between plasticity changes and compensatory mechanisms, also considering the different cognitive profile in highly malignant and lower grade gliomas.

### 4.5. Open Issues

Brain tumor grades critically influence the dynamic malleability of the brain, so that neuroplasticity decreases in parallel to increased malignancy. Evidence of divergent compensatory pathways for slow and acute insults has been also confirmed by the literature on stroke, comparing the acute and chronic cortical rearrangements after the critical event [70]. Low-grade tumors more likely lead to functional plasticity, according to the observation that slow-growing lesions may induce cerebral reorganization processes because the recruitment mechanism of both ipsilateral and contralateral regions is more efficient. These reorganizations account for the relative preservation of cognitive functionality of LGG patients and can explain why a better functional recovery is achieved [11, 71]. In contrast, rapidly growing high-grade tumors lead to neurological deficits and cognitive disturbances [72, 73], suggesting a theoretically less efficient mechanism of compensation.

Other tumor-related features, such as tumor size, emerged as relevant in affecting brain plasticity in LGG and HGG. Small tumor volume as well as sharp radiologically defined tumor borders was found in patients with a high level of plastic rearrangements, during repeated awake surgeries [50, 51]. Apart from tumor-related characteristics, other factors that may interfere with the functional cerebral activity, and consequently on plasticity process, comprise anticonvulsants and adjuvant therapy (chemotherapy and radiotherapy), frequency of seizures [74, 75], and subjects' related characteristics (i.e., age), as showed in stroke patients [76].

### 4.6. Final Considerations in the Study of Brain Plasticity

In summary, significant suggestions for the investigation of cortical brain plasticity in adult patients affected by gliomas can be derived from this literature review. Firstly, the combination of the fMRI technique with intraoperative awake DES seems to be an effective strategy to describe short-term and long-term cortical rearrangements within subjects. A comprehensive protocol should include task-based fMRI as a noninvasive imaging technique to study cortical plasticity, with the exception of those patients in which neurovascular uncoupling is detected [56]. In these cases, MEG imaging may replace fMRI mapping. Currently, no evidence is available concerning the use of resting-state fMRI. However, in the future, we reasonably expect that also this task-free technique will be exploited for the study of cortical plasticity by assessing functional connectivity.

Moreover, neuropsychological evaluation of all relevant cognitive domains should be performed both at the diagnosis and during the follow-up, in order to identify a baseline and consequently monitor cognitive changes at different defined time points [77]. Longitudinal evaluation with noninvasive imaging and neuropsychological testing could be conducted starting from the very early phase and in a temporal window of about 3 to 6 months postsurgery.

Besides all tumor patients undergoing neurosurgical interventions affecting eloquent areas, the neuropsychological assessment may be extremely beneficial in particular for those presenting with infiltrative LGG. Indeed, since the extent of resection and the radicality of surgery highly affect their prognosis, the measure of neurocognitive functioning is of key importance in these patients.

Functional MRI appears to be a more suitable technique to study intra- and interhemispheric patterns of cortical rearrangements for language function, while n-TMS may be used as an alternative to fMRI to study intrahemispherical motor function reorganization. Patient-tailored neurosurgical strategy can be also better achieved by integrating functional imaging techniques and intraoperative mapping data, especially in repeated surgeries [78]. Since it would be difficult to study patients without any surgical treatment during their clinical course, given that tumor removal improves the overall survival, surgery should be always taken into account in plasticity evaluation.

Additionally, prospective study on cortical brain plasticity should include a sufficiently large sample of patients, in order to compare homogeneous subgroups with similar clinical characteristics or tumor-related features (i.e., cognitive profile or tumor's location or grade).

To conclude, detailed information regarding patients' treatment (chemotherapy, radiotherapy, and anticonvulsants) should be registered.

## 5. Conclusions

This review attempts to give insights into cortical mechanisms underlying neuroplasticity in glioma patients. A number of relevant methodological issues affecting the generalization of these findings emerge from these studies. A crucial point consists in the current lack of standards and consensus regarding the definition of plasticity, the imaging methodology, and the behavioral measurement.

From the present review, it clearly appears that a consensus definition of what is currently considered cortical plasticity in brain tumor patients has not been reached yet, and this may reflect the complexity of such a multifaceted phenomenon. Nonetheless, a lack of a consensus regarding defined plasticity outcome measures is still far from being uniform. Another key aspect consists in the use of divergent methods of analysis to assess functional changes, which has prevented the possibility of determining consistent evidences. Ultimately, another important concern is represented by the absence of a standard evaluation of the neurocognitive functioning in brain tumor patients.

Apart from the mentioned limits, both preoperative and intraoperative mapping techniques demonstrate to be pivotal in delineating possible cortical rearrangements in glioma patients during their clinical history.

Longitudinal studies with a large sample of patients are needed to understand the association between functional changes and progression of disease, especially in lower grade gliomas. Furthermore, neuropsychological assessment should be inserted in the clinical routine, as long as it represents an objective standardized measure. Finally, understanding how cognitive functional recovery is mirrored by specific imaging modifications could be extremely relevant.

In the future, combined studies of neuroimaging and intraoperative mapping, along with a systematical neuropsychological assessment, could help to systematically understand and conclusively interpret all these open issues.

## Figures and Tables

**Table 1:** Summary of the selected studies.

Study	Technique and assessment	Plasticity measure	Pre/postsurgery examinations	Experimental design	Sample	Age (mean ± SD)	Sex (M; F)	Tumor classification	Tumor location	Hemisphere	Tumor volume (mean ± SD)	Extent of resection
Majos A. et al., 2017 [46]	Motor fMRI+clinical and motor assessment+DES	*T*-values+cluster size (M1; PMA; SMA)	Pre: 1 week Post: 3 months	WS-BS: 16 pt pre vs. 9 pt post; small tumors vs. large tumors	16 HGG	53.1 ± 13.1	5; 11	Glioblastoma	Premotor, motor cortex^∗^	14 L; 2 R	6 pt < 40 cm^3^; 10 pt > 40 cm^3^	Maximal (5) Subtotal (11)
Bryszewski B. et al., 2013 [45]	Motor fMRI+clinical, motor and neuropsychological assessment+DES	*T*-values+cluster size (M1; PMA; SMA)	Pre: 1 week Post: 3 months	WS: 20 pt pre vs. 14 pt post	20 LGG	37.5 ± 15.7	7; 13	—	Premotor, motor cortex^§^	15 L; 5 R	32.8 ± 20.8 cm^3^	Maximal (12) Subtotal (7) Partial (1)
Kristo G. et al., 2015 [47]	Language fMRI+clinical and neuropsychological assessment+DES	BOLD signal changes in the spatial pattern of language activations	Pre: until 25 days Post: until 126 days	WS-BS: pt pre vs. pt post; pt with left-sided gliomas vs. 6 right-side gliomas and vs. 8 HC	20 LGG	41.5 ± 8.78	12; 8	Astrocytoma (6) Oligoastrocytoma (1) Oligodendroglioma (13)	Frontal (13) Temporal (5) Parietal (2)	14 L; 6 R	17.8 ± 14.0 cm^3^ (resected volume)	—
Gębska-Kośla K. et al., 2017 [52]	Language fMRI+speech assessment+DES	*T*-values+cluster size (Broca and Wernicke); LI	Pre: - Post: 3 months	WS: pt pre vs. pt post; pre and post speech assessment	6 LGG 4 HGG	36.3 ± 10.3	5; 5	Glioblastoma (3) Astrocytoma (4) Oligodendroglioma (1) Ependymoma (1) Mixed glioma (1)	Frontal (5) Temporal (5)	All left	23.4 ± 18.2 cm^3^	—
Conway N. et al., 2017 [49]	n-TMS+motor assessment	Comparison for each muscle between HSs–CoGs shifts	Interval btw two mapping: 12.1 ± 10.4 m (range 3-42 m)	WS-BS: pt pre vs. pt post; anterior tumors vs. posterior tumors	6 LGG 16 HGG	49.6 ± 3.2	13; 9	Glioblastoma (10) Astrocytoma (9) Oligoastrocytoma (1) Oligodendroglioma (2) Mixed glioma (4)	Precentral gyrus (anterior and posterior)	11 L; 11 R	—	Maximal (14) Subtotal (8)
Barz A. et al., 2018 [48]	n-TMS+motor assessment	Comparisons for each muscle CoGs	Pre: 26.1 ± 24.8 months Post: 46.3 ± 25.4 months	WS-BS: pt pre vs. pt post and pt vs. HC	5 LGG 15 HGG	39.5 ± 13.2	13; 7	Glioblastoma (2) Astrocytoma (8) Oligoastrocytoma (3) Other (3)	Perirolandic region	7 L; 13 R	—	Maximal (11) Subtotal (5) Partial (4)
Picart T. et al., 2018 [50]	DES+clinical and neuropsychological assessment	Comparison of site displacement	Interval btw surgeries: G1: 5 years; G2: 4.1 years	WS-BS: pt at 1^st^ surgery vs. pt 2^nd^ surgery; remapping group vs. no remapping group	G1: 22 LGG; 1 HGG G2: 15 LGG; 4 HGG^●^	G1: 32.2 ± 7.8 G2: 30.9 ± 7.4	G1: 13; 10 G2: 12; 7	—	G1: frontal (15) Insular (3) Other (5) G2: frontal (13) Insular (3) Other (3)	G1: 6 L; 17 R G2: 4 L; 14 R; 1 bilateral	1st surgery: G1: 32.9 ± 19.1 G2: 46.4 ± 25.1 (ml) 2nd surgery: G1: 43.4 ± 38.9 G2: 51.9 ± 32.7	1^st^ surgery: G1 and G2: 94% 2^nd^ surgery: G1: 92% G2:88%
Southwell D. et al., 2016 [51]	DES+clinical and neuropsychological assessment	Changes in stimulation response (pos⟶neg or neg⟶pos)	Interval btw surgeries: 4.1 ± 2.1 years	WS: 18 pt evaluated intraoperatively at 2 craniotomy	20 LGG^○^	34.3 ± 8.9	12; 6	WHO grade II (17) WHO grade III (3)	Frontal (5) Temporal (4) Parietal (1) Insular (10)	Dominant hemisphere	57.4 ± 33.6 cm^3^	1^st^ surgery: 95%

Abbreviations: LGG: low-grade glioma; HGG: high-grade glioma; fMRI: functional magnetic resonance imaging; n-TMS: navigated transcranial magnetic stimulation; L: left; R: right; PMA: premotor area; SMA: supplementary motor area; PT: patients; HC: healthy controls; LI: lateralization index; HSs: hot spots; CoG: centers of gravity; WS: within-subjects; BS: between-subjects; G1: group 1; G2: group 2. ^∗^Tumor location in: PMA-SMA-M1 (8 pt), M1 (2 pt), posterior to postcentral sulcus, partially involving M1 (6 pt); ^§^PMA-SMA-M1 (13 pt), M1 (1 pt), posterior to post-central sulcus, partially involving M1 (6 pt). ^●^From an initial cohort of 73 cases, a group of 42 eligible patients was included in the study; ^○^from an initial cohort of 561 cases, a group of 20 eligible patients was included in the study.
